# Supplementary material for: Investigation into Red Emission and Its Applications: Solvatochromic N-Doped Red Emissive Carbon Dots with Solvent Polarity Sensing and Solid-State Fluorescent Nanocomposite Thin Films
Source: Molecules. 2023 Feb 12;28(4):1755. doi: 10.3390/molecules28041755 (PMC9960500; doi:10.3390/molecules28041755)
Supplement: Supplementary file 1 [file molecules-28-01755-s001.zip › molecules-2205283-supplementary.pdf]

Investigation into Red Emission and its Applications: Solvatochromic N-Doped Red Emissive Carbon Dots with Solvent Polarity Sensing and Solid-State Fluorescent Nanocomposite Thin Films

Justin B. Domena[1], Ermin Celebic[1], Braulio C.L.B. Ferreira[1], Yiqun Zhou[1], Wei Zhang[1], Jiuyan Chen[1], Mattia Bartoli[2], Alberto Tagliaferro[2], Qiaxian Johnson[3], Bhanu P.S. Chauhan[3], Victor Paulino[1], Jean-Hubert Olivier[1], Roger M. Leblanc[1,*]

Author Address

[1] Department of Chemistry, University of Miami, Coral Gables, FL 33146, USA

[2] Department of Applied Science and Technology, Politecnico di Torino, Italy

[3] Department of Chemistry, William Paterson University of New Jersey, 300 Pompton Rd, Wayne, NJ 07470, USA

[*]Correspondence: rml@miami.edu

Supporting Figures:

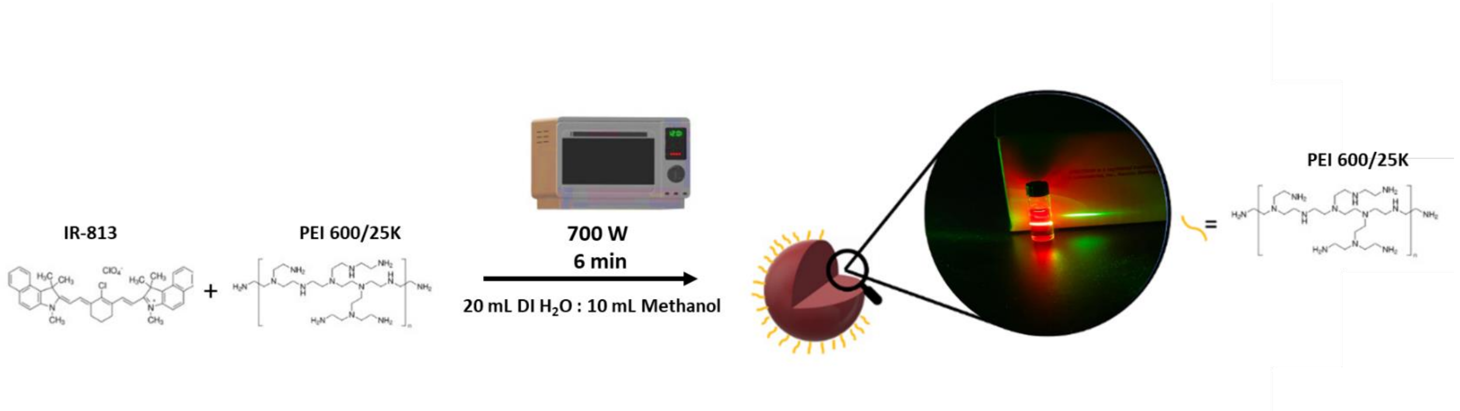

Figure S1: Graphic illustration of 813-CD synthetic procedure.

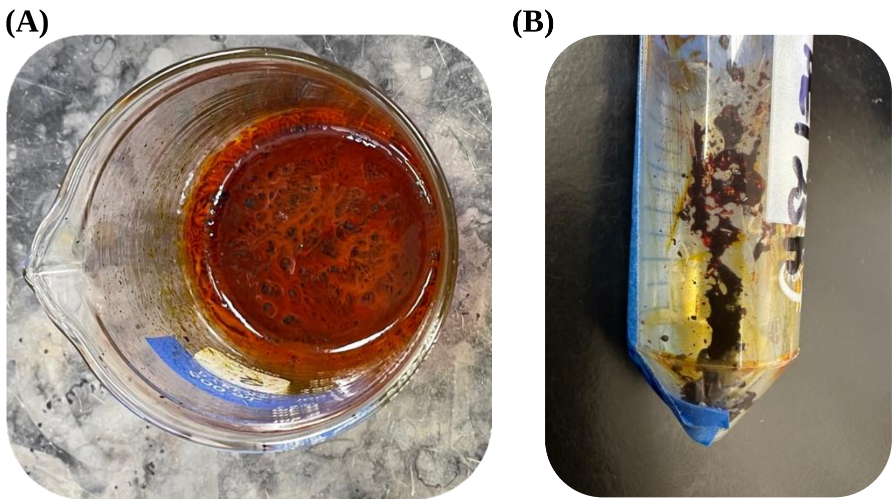

Figure S2: Images of 813-CDs post-synthesis and purification. (A) Image of 813-CD 600 PEI. (B) Image of 813-CD 25K PEI.

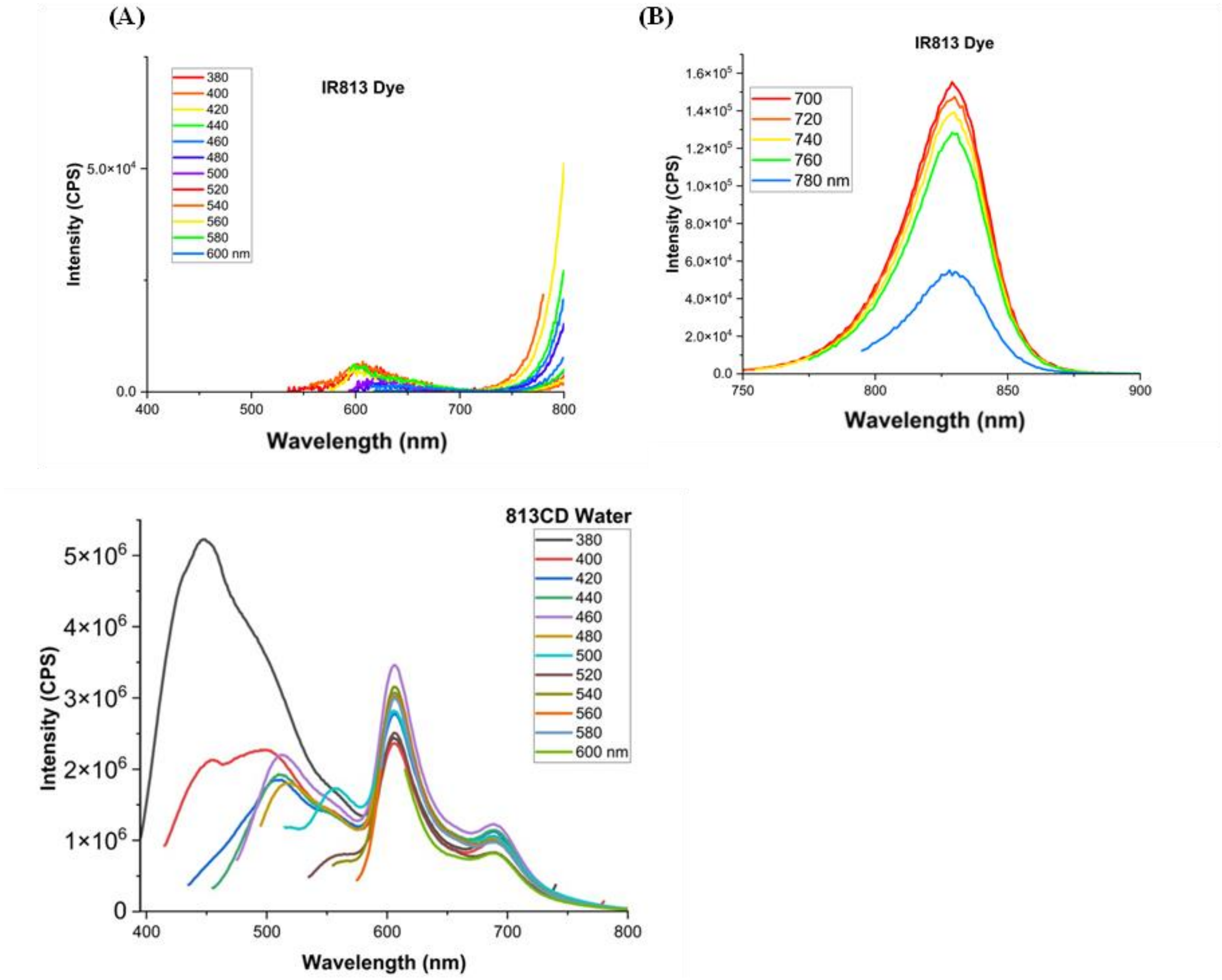

Figure S3: PL spectra of 813-CD versus IR-813. (A) PL spectrum of IR-813 from 400-800 nm in methanol. (B) PL spectrum of IR-813 from 750-900 nm in methanol. (C) PL spectrum of 813-CD from 400-800 nm in water.

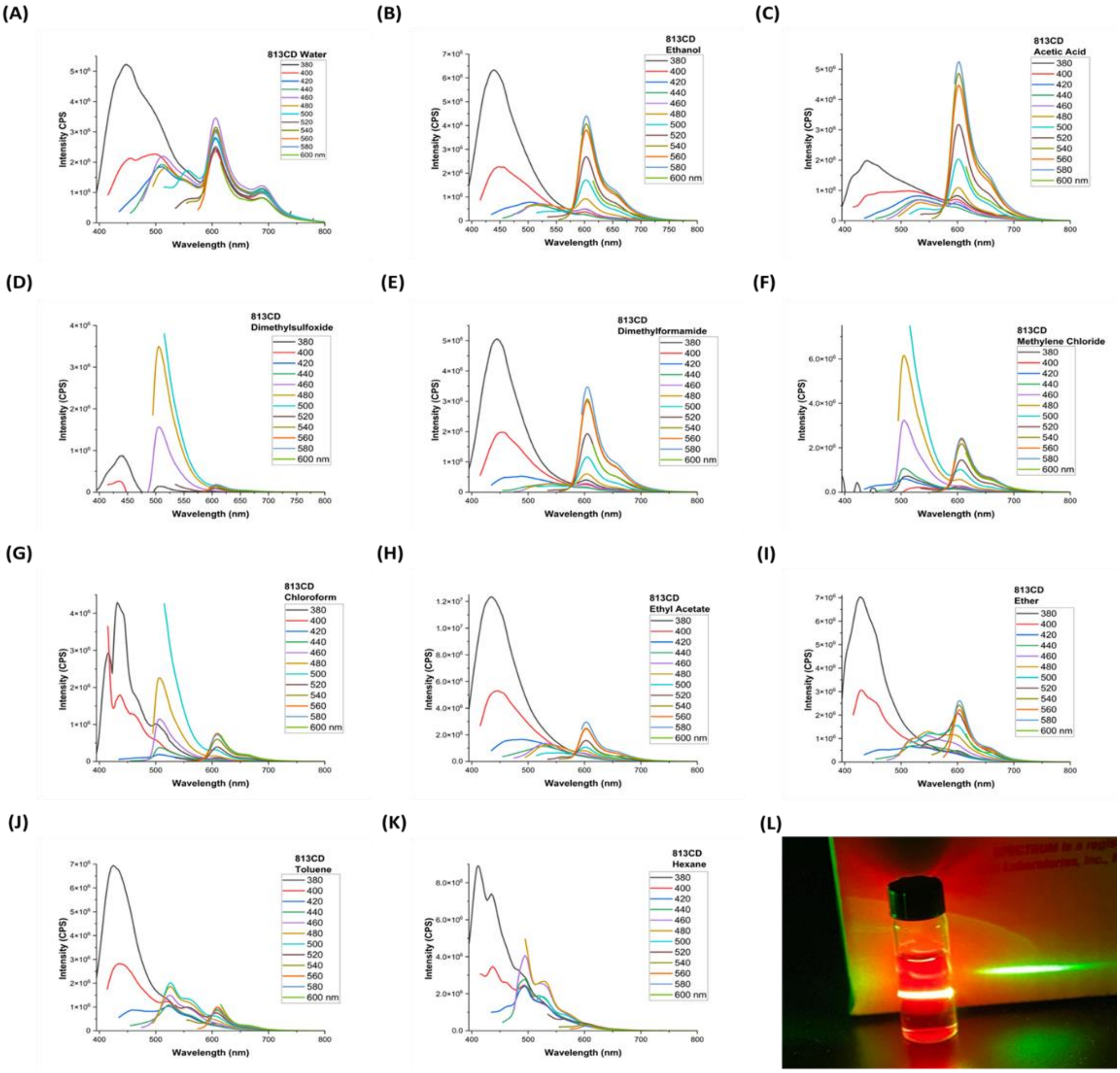

Figure S4. Optical analysis of 813-CDs in various solvents of decreasing polarities according to the solvent polarity index (*P*), PL spectrum. (A) water *P*:1.00, (B) ethanol *P*:0.654, (C) acetic acid *P*:0.648, (D) DMSO *P*:0.444, (E) DMF *P*:0.386, (F) methylene chloride *P*:0.309, (G) chloroform *P*:0.259, (H) ethyl acetate *P*:0.228, (I) ether *P*:0.117, (J) toluene *P*:0.099, and (K) hexane *P*:0.009, respectively.; (L) 813-CDs in water under excitation at 535 nm.

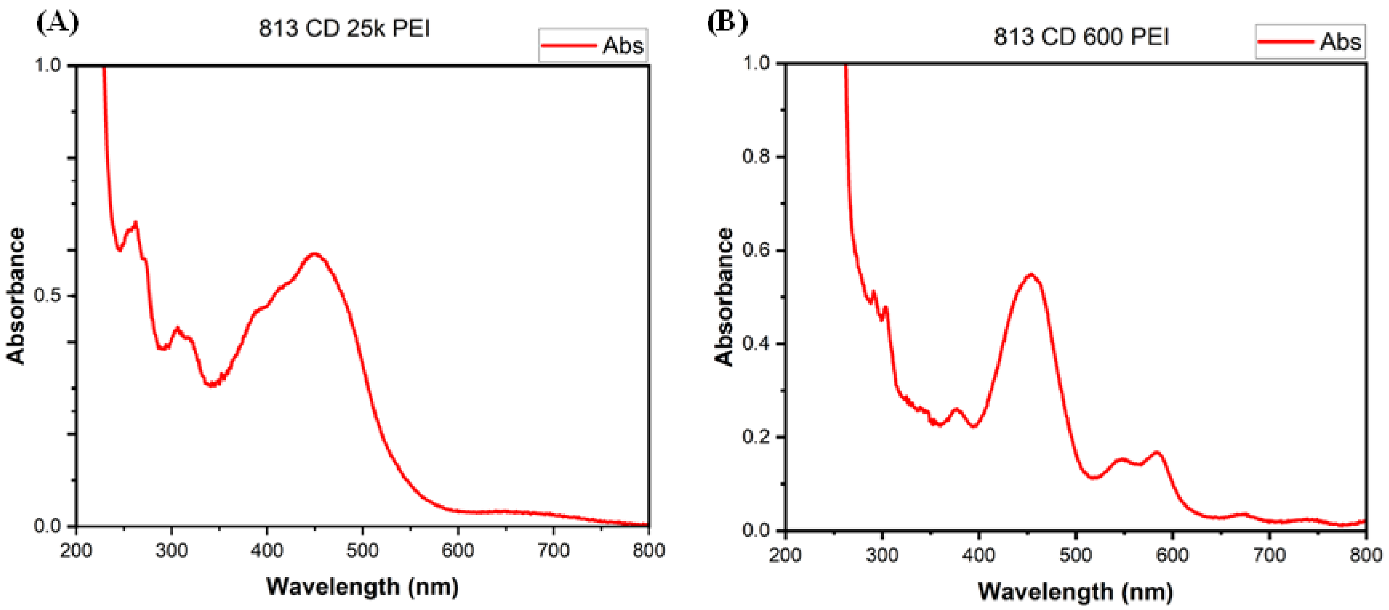

Figure S5: UV-vis absorbance spectra of 813-CDs in water. (A) UV-vis spectrum of 813-CD 25K PEI from 200-800 nm. (B) UV-vis spectrum of 813-CD 600 PEI from 200-800 nm.

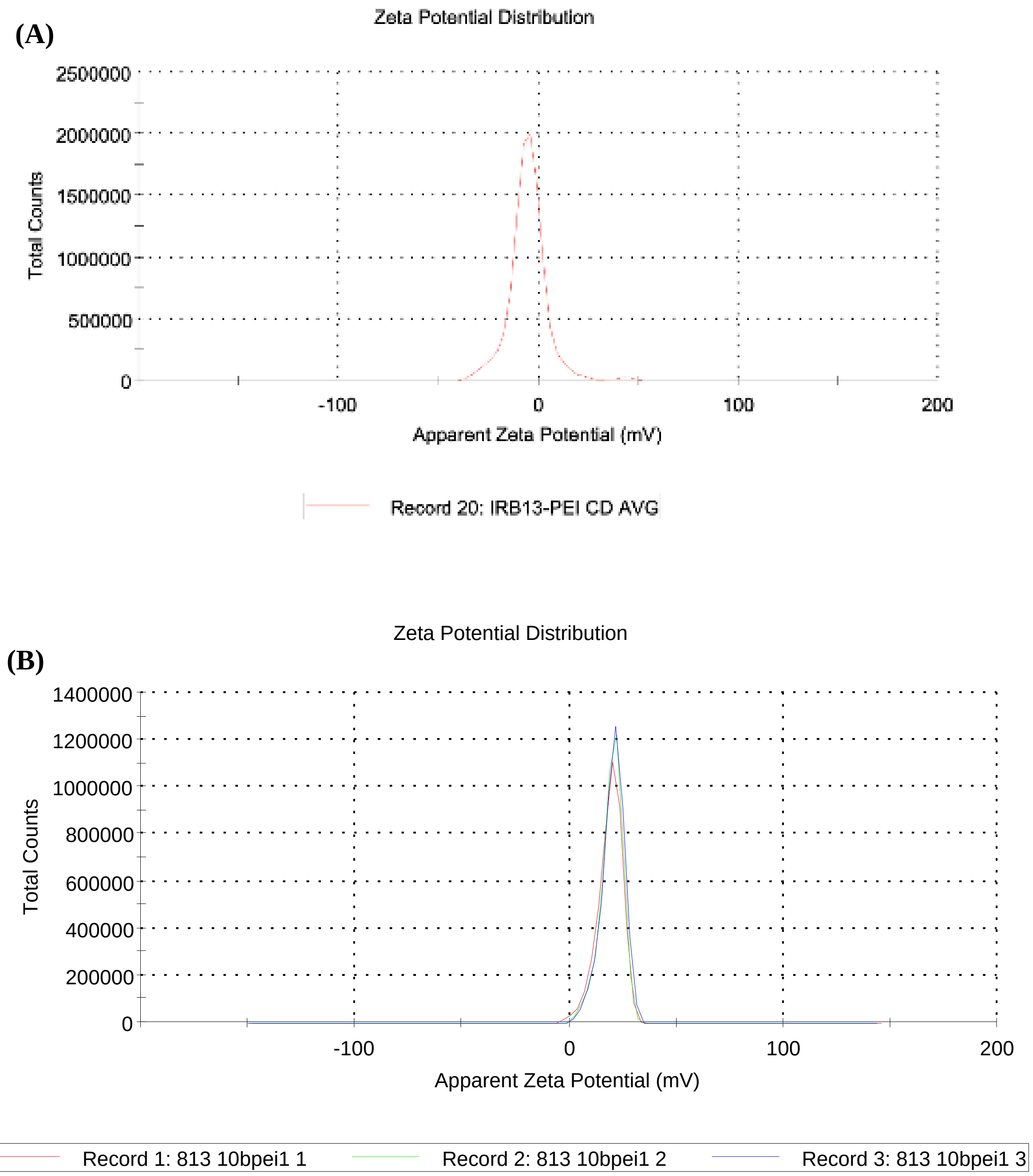

Figure S6: Zeta potential of 813-CDs. (A) Zeta potential of 813-CD 600 PEI, -5.11 mV. (B) Zeta potential of 813-CD 25K PEI, +18.8 mV

	Summary Chart of 813-CDs						
**CD**	**Dye**	**Polymer**	**Microwave (Watts)**	**Time (s)**	**Size (nm)**	**Farthest PL (nm)**	**Application**
813CD-600 PEI	IR-813	PEI- 600	700	360	6.0-6.5	698	PVP Film Composite
813CD-25K PEI	IR-813	PEI- 25 K	700	360	2.0-3.5	776	Positively Charged Surface

Table S1. Summary of CD methodology and properties.
